# Mechanisms of ion transport regulation by HNF1β in the kidney: beyond transcriptional regulation of channels and transporters

**DOI:** 10.1007/s00424-022-02697-5

**Published:** 2022-05-13

**Authors:** Lotte E. Tholen, Joost G. J. Hoenderop, Jeroen H. F. de Baaij

**Affiliations:** Department of Physiology, Radboud Institute for Molecular Life Sciences, Radboud University Medical Center, P. O. Box 9101, Nijmegen, 6500 HB The Netherlands

**Keywords:** HNF1β, Electrolyte disturbances, Transcriptional regulation, Kidney development, Apical-basolateral polarity

## Abstract

Hepatocyte nuclear factor 1β (HNF1β) is a transcription factor essential for the development and function of the kidney. Mutations in and deletions of *HNF1β* cause autosomal dominant tubule interstitial kidney disease (ADTKD) subtype HNF1β, which is characterized by renal cysts, diabetes, genital tract malformations, and neurodevelopmental disorders. Electrolyte disturbances including hypomagnesemia, hyperuricemia, and hypocalciuria are common in patients with ADTKD-HNF1β. Traditionally, these electrolyte disturbances have been attributed to HNF1β-mediated transcriptional regulation of gene networks involved in ion transport in the distal part of the nephron including *FXYD2*, *CASR*, *KCNJ16*, and *FXR*. In this review, we propose additional mechanisms that may contribute to the electrolyte disturbances observed in ADTKD-HNF1β patients. Firstly, kidney development is severely affected in *Hnf1b*-deficient mice. HNF1β is required for nephron segmentation, and the absence of the transcription factor results in rudimentary nephrons lacking mature proximal tubule, loop of Henle, and distal convoluted tubule cluster. In addition, HNF1β is proposed to be important for apical-basolateral polarity and tight junction integrity in the kidney. Interestingly, cilia formation is unaffected by *Hnf1b* defects in several models, despite the HNF1β-mediated transcriptional regulation of many ciliary genes. To what extent impaired nephron segmentation, apical-basolateral polarity, and cilia function contribute to electrolyte disturbances in HNF1β patients remains elusive. Systematic phenotyping of *Hnf1b* mouse models and the development of patient-specific kidney organoid models will be essential to advance future HNF1β research.

**Supplementary Information:**

The online version contains supplementary material available at 10.1007/s00424-022-02697-5.

## Introduction

Hepatocyte nuclear factor 1β (HNF1β) is a transcription factor expressed in epithelial tissues including the kidney, pancreas, liver, and genital tract and is essential for the development and function of these tissues [20, 22, 32, 33, 45, 90]. Within the kidney, HNF1β is expressed in all epithelial cells of the nephron and operates in homodimeric or heterodimeric complexes with HNF1α [20].

Mutations or deletions in *HNF1β* are responsible for a dominantly inherited, multisystem disease called autosomal dominant tubulointerstitial kidney disease type HNF1β (ADTKD-HNF1β) [27]. The disease was originally described as renal cysts and diabetes syndrome (RCAD), as kidney cysts (present in 60% of all patients) and maturity-onset diabetes of the young (MODY5) (40%) are common in patients with *HNF1β* defects [79]. However, the disease has a variable presentation, and not all patients suffer from cysts or diabetes. Kidney anomalies are often present and include renal hypoplasia, unilateral renal agenesis, microcystic dysplasia, and horseshoe kidney. As a consequence, kidney function is impaired in approximately half of the affected children and adults and progresses to end-stage renal disease in 12% of the patients [28, 57, 65]. In contrast to other cystic disorders, electrolyte disturbances are common in ADTKD-HNF1β patients [29, 49, 65]. In particular, the presence of hypomagnesemia is an important predictive criterium to suspect ADTKD-HNF1β [65]. Additionally, hypokalemia, hypocalciuria, hyperparathyroidism, and metabolic alkalosis are present in a minor group of patients [4, 10, 77, 79]. Extrarenal manifestations of ADTKD-HNF1β consist of diabetes, neurodevelopmental disorders, genital and urinary tract malformations, gout, and elevated liver enzymes [10, 12, 79].

The incidence of *HNF1β* defects is estimated to be 1:200,000 [91]. Approximately 150 different mutations have been reported [18]. These mutations can be familial with a dominant inheritance pattern (60%) or de novo (40%). The majority of the mutations are located in the first four exons encoding the dimerization domain and DNA-binding domains, which are required for binding of HNF1β to the genomic sequence 5′-TTAATNTTTAAC-3′ in promoter or enhancer elements [18, 86]. In addition to intragenic mutations, a 17q12 deletion spanning 15 genes, including *HNF1β*, accounts for 50% of the cases [19, 26]. Consequently, it is essential to perform an analysis of structural variants in the *HNF1β* gene, for instance by multiplex ligation-dependent probe amplification (MLPA).

Several groups have attempted to formulate diagnostic criteria to select patients for genetic *HNF1β* screening. Faguer and colleagues created an HNF1β score based on the clinical presentation [29]. However, several groups demonstrated that patients can be missed using the HNF1β score due to the variability in clinical presentation [18, 65]. The current KDIGO guidelines, therefore, use much simpler diagnostic criteria mainly based on the presence of kidney anomalies [27]. However, these criteria are often not specific for the HNF1β subtype of ADTKD and bear the risk of not identifying the patients that initially present with diabetes or electrolyte phenotype [26, 77]. Several groups have demonstrated that the presence of hypomagnesemia may be particularly predictive of *HNF1β* mutations [6, 65, 77].

In this review, we present the current knowledge on the electrolyte disturbances in ADTKD-HNF1β patients and discuss the possible mechanisms underlying these disturbances.

## Electrolyte disturbances in ADTKD-HNF1β patients

The introduction of next-generation sequencing in standard genetic diagnostic pipelines has resulted in the identification of thousands of ADTKD-HNF1β patients worldwide. Although ADTKD-HNF1β is a rare Mendelian disorder, these technological advances have allowed the formation of large cohorts of HNF1β patients [6, 26, 48, 55, 57]. Careful phenotyping of these cohorts has demonstrated that hypomagnesemia, hyperparathyroidism, hyperuricemia, and hypocalciuria are common in patients with *HNF1β* defects [5, 6, 30, 55, 92]. Only a minority of the patients have electrolyte disturbances including hypokalemia, metabolic alkalosis, and polyuria [6].

Hypomagnesemia (serum magnesium (Mg^2+^) < 0.7 mM) is the most common electrolyte disturbance in ADTKD-HNF1β patients. The penetrance of this symptom is estimated to range between 25 and 75% [5, 6, 29, 65, 77]. Several groups have aimed to explain the variability of reported hypomagnesemia cases among cohorts. Prospective cohort studies tend to report the presence of hypomagnesemia more often than retrospective analyses, indicating the poor implementation of Mg^2+^ measurements in the standard clinical blood biochemistry panels [77]. Several reports noted that young children have generally higher serum Mg^2+^ concentrations [6, 18, 77]. It was therefore proposed that hypomagnesemia developed later in childhood [6]. However, this notion was recently challenged by Kolbuc and colleagues [92]. Their detailed analysis demonstrated that serum Mg^2+^ levels are higher in early childhood in both HNF1β patients and healthy controls. Consequently, the reference range of 0.7–1.1 mmol/L is not applicable for young children, resulting in an underestimation of hypomagnesemia in early childhood. Studies establishing age- and gender-specific reference ranges are, therefore, needed.

Hyperparathyroidism (serum parathyroid hormone (PTH) > 6.5 pmol/L) was initially only described in single patients [5, 28]. However, systematic PTH measurements in small cohort studies demonstrated the presence of increased PTH levels in 80% of patients [30, 55]. Because PTH is not reported in many cohort studies, the exact percentage of ADTKD-HNF1β patients suffering from hyperparathyroidism is unknown. Especially, because small cohort studies bare the risk of selection bias, resulting in an overestimation of hyperparathyroidism [30, 55]. Of note, chronic kidney disease may contribute to elevated PTH levels on top of direct HNF1β effects.

Hyperuricemia (serum uric acid > 8 mg/dL) is present in 20–30% of all patients with ADTKD-HNF1β [48, 55, 57, 65]. Reduced kidney function is considered the main mechanism explaining hyperuricemia in ADTKD-HNF1β. Additionally, serum uric acid is independently associated with PTH levels, suggesting that PTH contributes to the molecular mechanism [92]. Indeed, PTH is known to inhibit uric acid secretion by downregulation of ATP-binding cassette transporter G2 (ABCG2) [74]. Interestingly, HNF1β also regulates the expression of renal urate transporter *URAT1* [39]. Nevertheless, hyperuricemia and hyperparathyroidism are poor predictors of *HNF1β* defects as it is also common in other forms of end-stage renal disease [65, 92].

Hypocalciuria is common in patients with ADTKD-HNF1β. The exact penetrance of hypocalciuria is unknown because the reference range for renal calcium (Ca^2+^) excretion has no generally established lower limit. Nevertheless, several studies demonstrated that urinary Ca^2+^ levels are significantly lower in patients with *HNF1β* defects compared to controls [5, 6].

Although serum potassium (K^+^) and bicarbonate (HCO_3_^−^) levels are poorly reported in ADTKD-HNF1β cohorts, Adalat and colleagues demonstrated that HNF1β patients have decreased serum K^+^ and increased serum HCO_3_^−^ levels, especially in late childhood [6]. Indeed, case reports have reported K^+^ values close to the lower border of the reference range (serum K^+^ 3.5–5.0 mM) [6, 28, 77]. Although these patients are not strictly hypokalemic, their serum K^+^ concentration is lower than in the general population.

The presence of hypomagnesemia, hypokalemia, metabolic alkalosis, and hypocalciuria is reminiscent of the phenotype of Gitelman syndrome [93, 94]. Indeed, the initial diagnosis of some patients has been Gitelman syndrome, until genetic investigations revealed mutations in the *HNF1β* gene [7]. However, it should be noted that renin–angiotensin–aldosterone system (RAAS) activation is scarce in patients with *HNF1β* defects, whereas it is a cardinal symptom of Gitelman patients. Moreover, hypertension is present in 22% of children with ADTKD-HNF1β, whereas Gitelman patients are generally hypotensive compared to healthy family members [69, 95]. Although it should be noted that chronic kidney disease in ADTKD-HNF1β patients may contribute to the hypertension phenotype.

## Mechanisms of disturbed electrolyte transport in ADTKD-HNF1β patients

The disturbed electrolyte transport caused by defects in *HNF1β* has classically been attributed to direct transcriptional regulation of key transporter genes along the nephron [79, 96]. In this review, we will provide an overview of the main transport mechanisms that are determined by HNF1β function. Moreover, we will consider additional mechanisms beyond direct transcriptional regulation, which may contribute to the ADTKD-HNF1β disease phenotype.

### Transcriptional control of transporters and channels

**Fig. 1:**
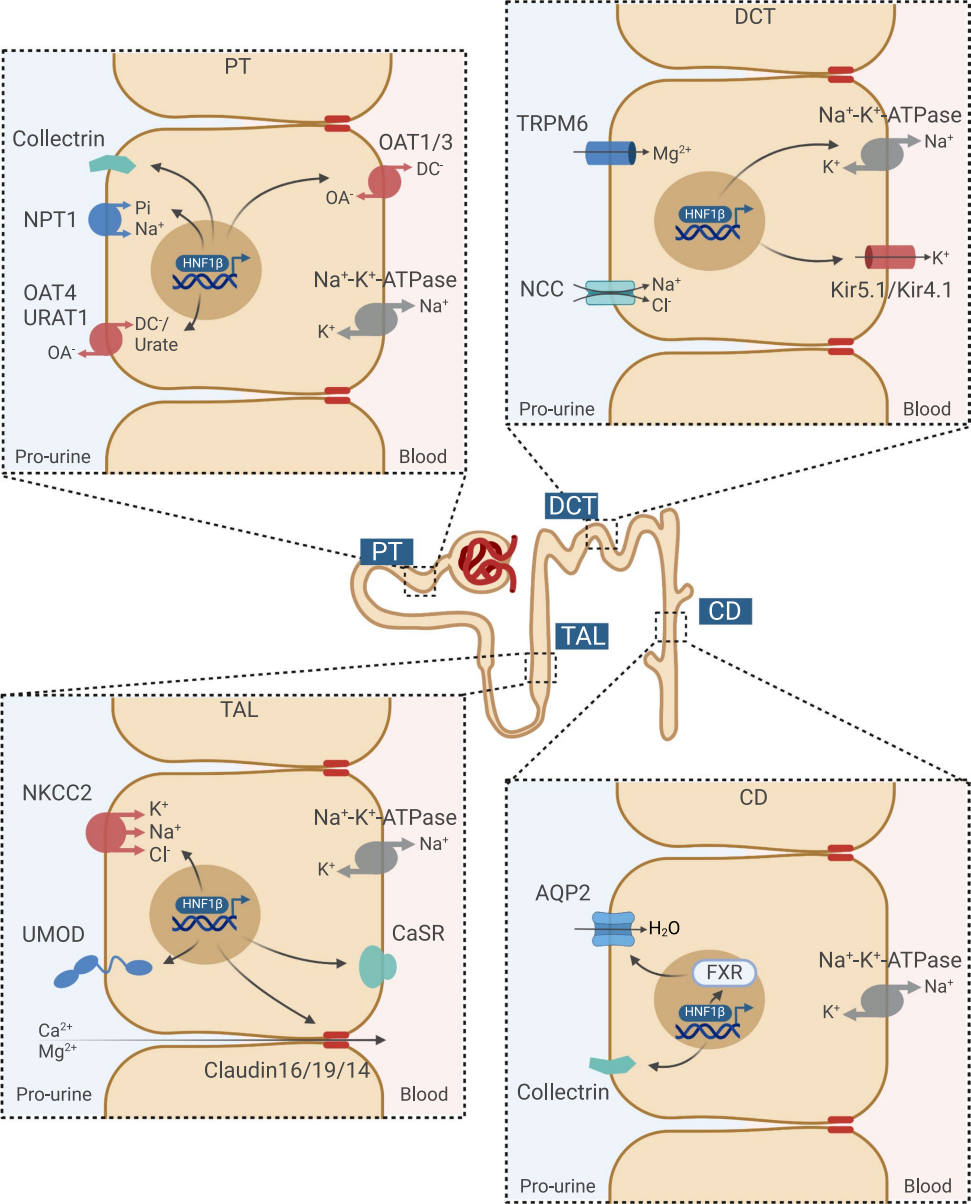
HNF1β regulates expression of channels, and transporters in all segments of the nephron. HNF1β regulates target genes involved in electrolyte handling in the PT including *TMEM27* encoding the amino acid transport regulator (Collectrin); *SLC17A1* encoding the Na-phosphate transporter 1 (NPT1); *SLC22A6*, *SLC22A8*, and *SLC22A11* encoding the organic anion transporters (OAT1, OAT3, OAT4); and *SLC22A12* encoding the renal urate transporter (URAT1); in the TAL including *SLC12A1* encoding the Na^+^-K^+^-2Cl^−^ co-transporter (NKCC2); *UMOD* encoding uromodulin (UMOD); *CASR* encoding the calcium sensing receptor (CaSR); and *CLDN16* encoding Claudin 16; in the DCT including *KCNJ16* encoding the subunit of the inward rectifier K^+^ channel (Kir5.1) and *FXYD2* encoding the Na^+^-K^+^-ATPase subunit gamma; in the CD including *TMEM27* and *NR1H4* encoding the farnesoid X nuclear receptor (FXR). In return, transcription factor FXR regulates expression of *AQP2* in the CD. *PT* proximal tubules, *DCT* distal convoluted tubule, *TAL* thick ascending loop of Henle, *CD* collecting duct, *OA*^−^ organic anion, *DC*^−^ dicarboxylate

The hypomagnesemia, hypokalemia, and hypocalciuria observed in ADTKD-HNF1β patients are generally assigned to distal tubule dysfunction. In the first description of electrolyte defects in ADTKD-HNF1β patients by Adalat and colleagues, *FXYD2* was identified as a transcriptional target in the distal convoluted tubule (DCT) (Fig. 1) [5]. *FXYD2* encodes the γ subunit of the Na^+^-K^+^-ATPase, and *FXYD2* mutations are causative for hypomagnesemia [23, 51]. In recent years, the cardinal role of the Na^+^-K^+^-ATPase was further demonstrated by the identification of *ATP1A1* mutations, encoding the α subunit of the Na^+^-K^+^-ATPase, as a cause of hypomagnesemia [67]. It has been hypothesized that reduced Na^+^-K^+^-ATPase activity in the DCT will result in depolarization of the basolateral membrane, resulting in an increased intracellular chloride (Cl^−^) concentration. Indeed, a high intracellular Cl^−^ concentration has been established to inhibit WNK kinases and thereby the phosphorylation and activity of the thiazide-sensitive Na^+^-Cl^−^ co-transporter (NCC). Clinical studies confirmed that ADTKD-HNF1β patients have a diminished response to thiazide, confirming lower NCC activity in patients [8]. Interestingly, NCC expression is also decreased in *Hnf1b* knock-out (KO) mice [41].

Moreover, HNF1β regulates the transcription of *KCNJ16*, which codes for the Kir5.1 subunit of the basolateral K^+^ channel in the DCT (Fig. 1) [41]. This Kir4.1/Kir5.1 K^+^ channel allows recycling of K^+^ to drive Na^+^-K^+^-ATPase activity. Uncoupling of this “pump-leak mechanism” will result in depolarization of basolateral membrane activity and reduced NCC activity by the same mechanisms as described above [97]. The importance of the Kir4.1/Kir5.1 channel was further established by the identification of *KCNJ10* and *KCNJ16* mutations in patients with hypokalemia and hypomagnesemia, mimicking Gitelman syndrome [13, 68, 98]. Nevertheless, hypokalemia and metabolic alkalosis are only present in a subset of patients with *HNF1β* defects, which is in line with the phenotype of patients with *FXYD2* or *ATP1A1* mutations [23, 67]. One might hypothesize that this phenotypic variability is explained by the degree of Na^+^-K^+^-ATPase dysfunction and the presence of compensatory effects.

The concomitant HNF1β-dependent regulation of basolateral Na^+^ and K^+^ transport by *FXYD2* and *KCNJ16* demonstrates that transcription factors generally regulate gene networks rather than single genes. Similarly, HNF1β determines a gene network controlling the urine concentrating ability of the kidney [2]. A collecting duct-specific *Hnf1b* KO mouse model showed a reduced urine osmolality [2]. RNA sequencing and ChIP sequencing identified 27 osmosensitive genes that are dependent on HNF1β binding [2]. Among the HNF1β targets is the farnesoid X receptor (FXR), which is essential for urine concentration by regulating aquaporin 2 (AQP2) expression (Fig. 1) [2, 88]. Indeed, apical plasma membrane expression of AQP2 is reduced in collecting duct cells expressing an *Hnf1b* mutant [2]. Interestingly, FXR directly activates the expression of Mg^2+^ channel *Trpm6* in mouse intestines [40]. Hence, HNF1β might indirectly regulate *Trpm6* expression in the intestines and kidneys through FXR, contributing to disturbed Mg^2+^ homeostasis in HNF1β patients.

Although HNF1β is also expressed in the thick ascending limb of Henle’s loop (TAL) and this segment transports substantial amounts of Na^+^, K^+^, Ca^2+^, and Mg^2+^, the role of HNF1β in electrolyte transport in this segment remains elusive. In the TAL, HNF1β was demonstrated to regulate the expression of *SLC12A1*, encoding the Na^+^-K^+^-Cl^−^ co-transporter 2 (NKCC2) (Fig. 1) [36]. As NKCC2 facilitates monovalent ion transport and provides the driving force for paracellular divalent cation transport, one would expect that downregulation of NKCC2 would cause major defects. Particularly, because the downstream DCT segment is affected as well and the compensatory capacity is therefore low. Nevertheless, features of TAL dysfunction such as polyuria, RAAS activation, hypercalciuria, and nephrocalcinosis are generally absent in ADTKD-HNF1β patients.

Several studies have demonstrated that HNF1β activates the expression of uromodulin (*UMOD*) and the calcium-sensing receptor (*CASR*) (Fig. 1) [32, 42]. As UMOD mutations are known to cause medullary cysts, this regulatory pathway may contribute to the cystic phenotype of patients with *HNF1β* defects. Reduced UMOD expression in ADTKD-HNF1β patients may also have implications for renal electrolyte handling since UMOD has been demonstrated to activate NKCC2, NCC, transient receptor potential melastatin type 6 (TRPM6), and TRP vanilloid type 5 (TRPV5) activity [54, 56, 75, 83]. However, as the CaSR is an important negative regulator of UMOD, *HNF1β* defects may simultaneously inhibit *UMOD* expression and release the inhibition by the *CaSR* [76]. Consequently, the reduced UMOD expression may be dampened.

The regulation of CaSR may be of particular importance in the parathyroid gland. CaSR activation in the parathyroid gland inhibits PTH release. The PTH promoter is repressed by HNF1β binding [30]. Hence, *HNF1β* defects directly increase PTH secretion. On top of that, reduced *CaSR* expression may also activate PTH secretion [42]. Indeed, ADTKD-HNF1β patients suffer from hyperparathyroidism [30, 55]. However, it should be noted that the in vitro experiments demonstrating the regulation of the *CaSR* promoter by HNF1β have been performed only in kidney cell lines and should be repeated in parathyroid models. Additionally, both increased PTH secretion and decreased renal CaSR expression are expected to raise calcium levels in the blood. Nonetheless, hypocalcemia is not consistently observed in ADTKD-HNF1β patients.

*HNF1β* is expressed in all tubule segments of the nephron [20]. Consequently, transcriptional targets of HNF1β have also been identified in the proximal tubule (PT). The expression of organic anion transporters (OAT1, OAT3, OAT4), the Na^+^-phosphate transporter 1 (NPT1), and the renal urate transporter (URAT1) is regulated by HNF1β (Fig. 1) [37–39, 66, 99]. Nevertheless, only a few individual cases were presenting with Fanconi syndrome, suggesting relatively mild PT dysfunction [28]. The absence of a PT phenotype in most patients can potentially be explained by the action of HNF1α, which may compensate for the loss of HNF1β in this segment. As HNF1α is within the kidney exclusively expressed in the PT, other nephron segments do not benefit from this compensatory action [100]. Altogether, systematic studying of HNF1β binding sites in the kidney has resulted in the identification of many genes that are transcriptionally regulated by HNF1β [1, 2, 16, 41, 42]. To date, most studies have investigated HNF1β function by measuring the promoter activity of isolated genes using promoter-luciferase assays. Although these artificial overexpression systems have been instrumental to detect the most prominent regulatory pathways, gene transcription also largely depends on chromatin modifications, the presence of co-activators/co-repressors, or post-translational modifications that are not captured by promoter assays. The recent advances in single-cell genomics and proteomics will allow us to further decipher transcriptional regulation by HNF1β beyond individual genes, by analyzing gene networks and combining -omics approaches.

### The role of HNF1β in ureteric bud branching and nephron patterning during kidney development

HNF1β has an essential role during kidney development [20, 32, 90]. The developmental defects may contribute to electrolyte disturbances observed in patients with ADTKD-HNF1β. In Gitelman syndrome, impaired DCT development has been postulated as one of the main causes of Mg^2+^ wasting [97]. Consequently, defects in kidney tubule patterning should be considered when studying the molecular pathogenesis of ADTKD-HNF1β. Various kidney-specific or inducible mice models have been generated over the past years to determine the role of HNF1β in kidney development (Table 1).

**Table 1:** Systematic comparison of all published *Hnf1b* mouse models

Mouse model			Electrolytephenotype	Developmentaldefects	Presence of cysts				Apico-basolateral polarity	Renal function	Survival	Other	Reference
Tissue	Geneticmodel	Promoter			Cortex	Medulla	Tubular	Glomerular					
Kidney	KI Dominant negative *Hnf1b*	*Cdh16*	NR	NR	+	+	+	+	NR	Normal to increased BUN levels	NR	NR	[106]
Kidney	KO Cre-loxP	*Cdh16*	NR	Abnormalities of mature nephrons	+	+	+	+	Similar number of cilia	Increased serum and urea creatinine	P10–P21 (75%)	Hydronephrosis (92%) Interstitial fibrosis (NR)	[32] [16]
Full body	Inducible KO at P1 MxCre-LoxP	-	NR	NR	+	+	+	NR	NR	NR	NR	Hydronephrosis (NR)	[78]
Full body	Inducible KO at P10 MxCre-LoxP	-	NR	NR	-	-	-	-	NR	NR	NR	-	[78]
Full body with exception of ExEn	KO Tetraploid aggregation	-	NR	Delayed and defective UB branching Absence of MET and fewer MM condensations	NR	NR	NR	NR	NR	NR	NR	Hypoplasia (100%)	[46]
MM	KO Cre-loxP	*Six2*	NR	Absence of bulge in S-shaped body Rudimentary nephrons^a^	+	-	-	+	NR	NR	P0–P2	Hydronephrosis (15%)	[50]
Nephron progenitors	KO Cre-loxP	*Wnt4*	NR	Absence of bulge in S-shaped body Rudimentary nephrons^a^ Fewer glomeruli	+	-	-	+	Correctly polarized RVs	NR	P0–P2	Hydronephrosis (occasionally) Hypoplasia (NR)	[35]
Nephron progenitors	HET KO Cre-loxP	*Wnt4*	NR	NR	+	+	+	+	NR	NR	Normal	Hydronephrosis (occasionally)	[35]
CD	KO Cre-loxP	*Pkhd1*	Reduced urine osmolality Decreased Na^+^, K^+^, and urea urine concentrations	NR	+	+	+	+	NR	Increased serum creatinine	Normal	Polyuria (NR) Hydronephrosis (16–100%)^b^ Interstitial fibrosis (44–100%)^c^	[3]
UB	Mosaic KO Cre-loxP	*HoxB7*	NR	Defective UB branching and CD differentiation	-	+	+	-	Abnormal Fewer cilia	NR	P2 to P15	Hypoplasia (100%)	[25]
Full body	HET Splice-site mutation intron-2	-	Reduced urine osmolality Increased total Mg^2+^, Na^+^ and K^+^ urine excretion^d^ Increased urine Ca^2+^	Delayed PT differentiation Fewer glomeruli	+	+	+	+	Abnormal Fewer cilia	Normal plasma creatinine levels	P1 to P25 (10–15%)^e^	Hydronephrosis (33%)^e^ Duplicated kidney (17%)^e^ Polyuria (NR)	[103]

*KI* knock-in, *BUN* blood urea nitrogen, *NR* not reported, *KO* knock-out, *ExEn* extra-embryonic endoderm including visceral endoderm, *UB* ureteric bud, *MET* mesenchymal-epithelial transition, *MM* metanephric mesenchyme, *RV* renal vesicle, *HET* heterozygote, *CD* collecting duct, *PT* proximal tubules

^a^Nephron comprising a glomerulus connected to the collecting system by a short tubule displaying distal fates

^b^Age P7 and age > P35

^c^Age P35 and age > P35

^d^< 12 months of age

^e^In the C57BL/6 N background but not in 129/sv background

**Fig. 2:**
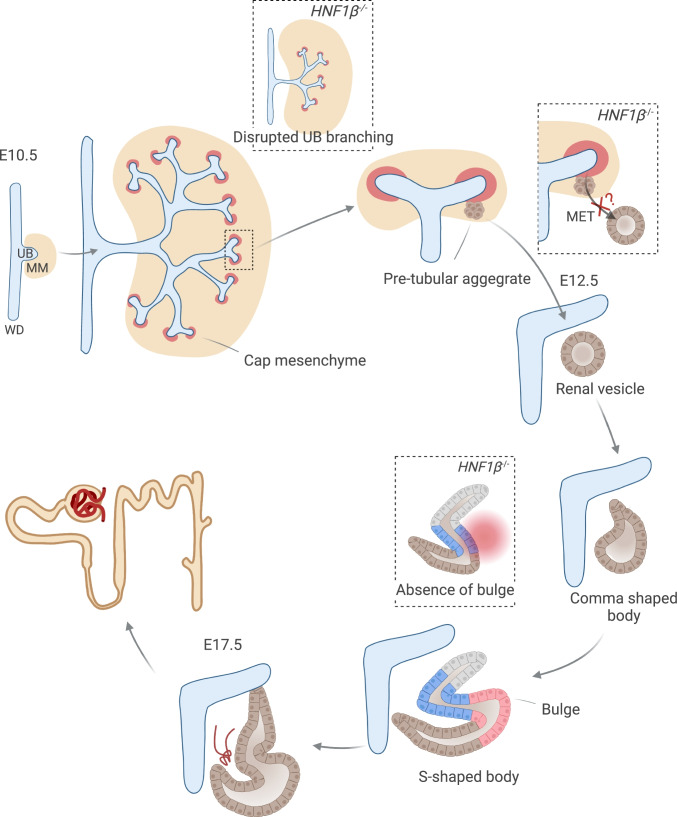
HNF1β is required for UB branching and nephron segmentation. Schematic representation of different stages of mouse metanephric nephron development. At E10.5, kidney development starts with the outgrowth of the UB into the MM. HNF1β is essential for normal branching of the UB that eventually will form the collecting duct system. Around E12.5, cells of the cap mesenchyme polarize into pretubular aggregates that will form renal vesicles which require MET. Whether HNF1β is involved in this early stage of nephrogenesis is not yet conclusive. Subsequently, renal vesicles differentiate into comma and S-shaped bodies. *Hnf1b* KO mice develop S-shaped bodies that lack the epithelial bulge that will give rise to the proximal and Henle’s loop tubule in the WT situation. Eventually at E17.5, part of the S-shaped body will associate with capillaries to form the glomerulus and other parts will form the nephron tubule. *WD* Wolffian duct, *UB* ureteric bud, *MM* metanephric mesenchyme, *MET* mesenchymal-epithelial transition

Mice with heterozygous *Hnf1b* null mutations have no phenotype, while complete deletion of *Hnf1b* in a mouse model is embryonically lethal due to its crucial role in embryonic visceral endoderm formation [21, 90]. Around E10.5, the development of the kidney starts with the outgrowth of the ureteric bud (UB) from the Wolffian duct (WD) into the metanephric mesenchyme (MM) (Fig. 2). The UB undergoes branching morphogenesis to form the collecting duct system and ureter, after which MM cells surrounding the tips of the ureteric branches form cap mesenchyme. Triggered by signals from the UB tips, these cap mesenchymal cells will polarize into primitive epithelial spheres (pretubular aggregates) to form the renal vesicles. Renal vesicles differentiate into comma- and S-shaped bodies; eventually, part of the S-shaped body will associate with capillaries to form the glomerulus, and other parts will form the nephron tubule that will connect to the collecting duct system. This tightly regulated process called nephrogenesis determines the development and segmentation of the kidney tubule. Although kidney development in humans and mice is very similar at a macroscopic level, organization (e.g., numbers of nephron progenitors and UB tips in human kidneys are increased compared to mice kidneys), timing, and gene expression patterns differ [44]. Therefore, extrapolating data obtained from mice to humans should be done with caution.

In early kidney development, *Hnf1b* is expressed in the WD and UB [46]. Whereas it is expressed during all nephrogenesis steps including the renal vesicle and comma- and S-shaped body, it is not expressed in the cap mesenchyme [46, 50]. Inactivation of *Hnf1b* in the mouse UB led to a massively mispatterned ureteric tree network along with defective collecting duct differentiation and polarization (Fig. 2) [25]. Moreover, using constitutive inactivation of *Hnf1b* in the epiblast by tetraploid aggregation, researchers show that HNF1β is required for UB branching and timing of outgrowth as well as WD maintenance [46]. Although most kidney development studies have been conducted in mouse models, recently heterozygous *HNF1β* KO (*HNF1β*^+*/*−^) ureteric bud organoids derived from human-induced pluripotent stem cells (iPSCs) were developed [101]. Wild-type (WT) ureteric bud organoids were polarized, had clear tubular lumen, and showed repeated branching morphogenesis [101]. Similar to *Hnf1b* KO mouse models, human *HNF1β*^+*/*−^ organoids showed loss of apical-basolateral polarity and had reduced numbers of budding regions [101].

In addition, several studies uncovered an important role for HNF1β in early nephron segmentation, more specifically in the development of the PT and TAL. HNF1β is required for the formation of a specific mid-limb subcompartment of the S-shaped body, the so-called epithelial bulge, that gives rise to the TAL and the PT (Fig. 2) [35, 50]. In mice, the absence of *Hnf1b* in the MM resulted in S-shaped bodies without the epithelial bulge and led to the development of nephrons characterized by dilated glomeruli directly connected to collecting ducts via short, primitive tubules displaying early distal markers [50]. Likewise, conditional inactivation of *Hnf1b* in nephron progenitors results in a reduction of tubular structures with a drastic decrease in PT clusters, medullar Henle’s loop tubules, and DCTs in kidneys from newly born mice (P0) [35]. Expression levels of Notch signaling molecules were strongly decreased in these mice, which may explain the lack of proximal-intermediate nephron segment fate acquisition [35, 50]. In line with these findings, expression of early PT (Hnf4a, Cubn, and Lrp2), mature PT (LTA), TAL (Slc12a1), and DCT (Pvalb) markers was drastically decreased in kidneys of mutant pups at P0 [35, 50]. Mutant S-shaped bodies may express early distal markers, but fail to differentiate into mature distal tubules [35]. Although HNF1β is important for early nephrogenesis, it is still unclear if it also plays a role during the initiation stage that requires mesenchymal-epithelial transition of the MM. In particular, inactivation of *Hnf1b* in the MM or in nephron precursors resulted in correctly polarized renal vesicles, indicating that HNF1β is not required to initiate nephrogenesis [35, 50]. In contrast, decreased numbers of pretubular aggerates were observed in *Hnf1b*-deficient mouse kidneys potentially caused by decreased levels of Wnt9b required for mesenchymal-to-epithelial transition underlying the initiation of nephrogenesis (Fig. 2) [46].

Comparable to the mice models, human iPSC-derived organoids with *HNF1β* KO formed podocytes and GATA3 + distal nephron segments but lacked cells expressing of PT (*LRP2, HNF4α*) and TAL markers (*UMOD*, *SLC12A1*) [64]. These findings are concomitant with a statistical overrepresentation of HNF1β-binding sites in the promoters of PT-specific genes [14, 102]. Altogether, these findings suggest that HNF1β is essential for UB branching and nephrogenesis and particularly affects the PT and TAL segments.

As KO mice models may not represent the effects of human mutations, Niborski et al. generated a mouse model introducing a human splice site mutation (< IVS2nt + 1G > T) [103]. Their mouse model displayed delayed PT differentiation, hydronephrosis, and cysts. Consistent with other mice models, PT markers were decreased from E14.5 to E17.5; however, S-shaped bodies appeared normal and PT maker expression was restored at P0 [103]. Interestingly, at 6 but not 12 months of age, *Hnf1b* mutant mice exhibited a reduced ability to concentrate urine associated with hypercalciuria but no hypomagnesemia or hyperkalemia was observed [103]. These findings suggest that HNF1β dysfunction in development may be compensated for at a later age.

How do these developmental defects translate to the electrolyte defects in the adult kidney? Remarkably, PT defects are rare in ADTKD-HNF1β, which is difficult to match with maldevelopment of the PT [28]. However, it should be noted that kidney development has been mostly studied in mice. In addition, PT defects could be compensated for by HNF1α transcriptional activity in postnatal life, as evidenced by partial restoration of several PT markers in adult kidneys of mice with a heterozygous splice site mutation in *Hnf1b* [103]. The impact of heterozygous mutations on kidney development in humans is largely unknown. Histological analysis of a limited number of cystic kidneys from human fetuses carrying *HNF1β* mutations showed defective or delayed nephrogenesis characterized by a decrease in nephron structures labeled by either LTA, NKCC2, or UMOD [11, 34, 47]. How and to what extent, developmental abnormalities in mice and humans, in particular the rudimentary nephrons lacking mature PT, TAL, and DCT observed in mice models, influence ion transport in adults is unknown. In recent years, an impressive number of human kidney organoids models have been generated and successfully employed to improve our understanding of kidney diseases (reviewed in [104]). Hence, organoid models may provide a valuable tool to better understand the role of HNF1β in human kidney development and electrolyte transport using relevant genetic models instead of full KOs.

### The role of HNF1β in apical-basolateral polarity, tight junction integrity, and primary cilia

Apical-basolateral polarity and tight junctions are key regulators of controlled water and ion movement in the kidney epithelium [24, 73]. Moreover, the primary cilium influences renal electrolyte transport in response to changes in tubular flow [52, 63, 72, 81]. In the following part of this review, we will discuss the proposed role of HNF1β in apical-basolateral polarity, tight junction function, and primary cilia development.

#### Apical-basolateral polarity

Apical-basolateral polarity allows the distribution of channels and transporters to distinct membrane domains and is critical for directional transport of ions and water from the pro-urine to the blood and vice versa [73]. Several polarity markers show aberrant localization or expression during kidney development in HNF1β mutant mice models [25, 103]. For instance, removal of *Hnf1b* from the UB in mice results in reduced expression of polarity markers *Cdh16* and *Pkhd1* in UB epithelium [25]. Moreover, in mice with a heterozygous splice site mutation in *Hnf1b*, decreased levels of HNF1β appear to disturb basal membrane organization without affecting apical cell polarity markers [103]. Interestingly, NKCC2 expression in TAL cells, normally apically expressed, was normal in non-cystic tubules, but the expression was downregulated in cystic tissue [103]. Studies performed by our group using an immortalized mouse collecting duct cell line with disrupted HNF1β function demonstrated a decrease in cell height compared to cells expressing WT HNF1β (unpublished data). Apical-basal growth is a characteristic of polarizing epithelia; likewise, studies using different types of epithelial cells have shown that a loss of cell integrity is associated with a decrease in cell height [59, 71]. In addition, *HNF1β*^+*/*−^ ureteric bud organoids derived from human iPSCs display loss of apical-basolateral polarity shown by reduced mRNA expression of apical markers, villin-2 (*EZRIN*) and protein kinase C zeta type (*PRKCζ*) [101]. Consistent with this putative role for HNF1β in establishing cell polarity, HNF1β-binding site motifs are enriched in ATAC-sequencing peaks and promoters of upregulated genes during in vitro 3D spheroid formation [105]. Together, this suggests that gene activation by HNF1β is important for cells to establish cell polarization.

#### Tight junction integrity

Tight junctions establish a border between the functionally different apical and basolateral membrane and act as a barrier for paracellular transport of water and ions [24, 89]. These structures contain a wide variety of proteins (occluding, claudins, junctional adhesion molecules) that define the permeability characteristics of epithelia [24, 58]. Structurally, Desgrange et al. showed that tight junctions appeared well-organized in the UB tips of developing *Hnf1b* mutant kidneys; however, lateral cell–cell junctions were irregular and the space between cells was larger [25]. Both disruptions in Ca^2+^ and Mg^2+^ homeostasis are frequently observed in ADTKD-HNF1β patients. Our unpublished data in immortalized cells showed a significant decrease in transepithelial resistance (TEER) values, a measure of paracellular pathway resistance involving tight junction integrity, in cells with disrupted HNF1β function compared to cells expressing WT *Hnf1b*.

#### Primary cilia development

HNF1β regulates an impressive number of genes that localize to the primary cilium including *PKHD1*, *PKD1*, *PKD2*, *IFT88*, *KIF12*, *CYS1*, and *PDE4C* (reviewed in [70]). Consequently, ciliary defects have been widely considered as the main cause of cyst formation in ADTKD-HNF1β patients [32, 70]. Nevertheless, it is unclear whether HNF1β is directly involved in primary cilium formation, despite the direct transcriptional activation of cilia genes. Two independent studies observed a decrease (25% and not quantified, respectively) of cilia in the cystic epithelium of developing mutant mice compared to WT mice [25, 103]. However, a different study observed normal cilia in cystic tubular cells compared to WT cells of mice with kidney-specific inactivation of *Hnf1b* (not quantified) [32]*.* Furthermore, humans and mice with *HNF1β* deficiency do display an absence of normal primary cilia in the bile duct.

The role of HNF1β in cilia function may also be relevant for electrolyte transport. The cilium acts as an antenna to sense tubular flow and converts changes in tubular pressure into signals that affect electrolyte transport along the nephron [52, 63, 72, 81]. Evidence for the involvement of cilia in flow sensing is based on the fact that flow-sensitive proteins polycystin 1 and transient receptor potential cation channel vanilloid-type 4 (TRPV4) localize to the primary cilium [43, 84, 87]. Furthermore, several examples demonstrate the putative importance of cilia in flow-mediated electrolyte transport. For instance, mice without ciliated TAL cells have diminished Na^+^ excretion in response to increased water intake causing differences in tubular pressure [72]. In addition, the removal of cilia in immortalized mouse DCT cells reduced transepithelial Ca^2+^ transport [52]. Additional quantitative studies and the use of high-resolution microscopy techniques to visualize key ciliary proteins should clarify whether HNF1β is involved in cilia function in the kidney.

The importance of cell polarity and tight junction integrity in ion homeostasis has been recognized for decades. Even though the analyzed studies demonstrate that *HNF1β* defects disturb apical-basolateral cell polarity and tight junction integrity, these mechanisms have never been considered in the pathogenesis of electrolyte disturbances observed in ADTKD-HNF1β patients [25, 103, 105]. Although many Hnf1b animal models have been developed, electrolyte disturbances and polarity defects are often not measured (Table 1). Systematic analysis of apical-basolateral polarity markers and intracellular signaling pathways may help further elucidate the role of cell polarity in electrolyte homeostasis.

### Additional pathways

Our literature review has demonstrated that several mechanisms contribute to electrolyte disturbances in patients with *HNF1β* defects. Nevertheless, it cannot be excluded that additional factors influence ion transport in these patients.

Firstly, the presence of cysts in the kidneys of ADTKD-HNF1β patients can lead to electrolyte disturbances, as observed in patients with autosomal dominant polycystic kidney disease (ADPKD) [60, 62]. Interestingly, the deletion of a transcriptional target of HNF1β and frequently mutated gene in ADPKD patients, called *Pkd1*, caused aberrant Mg^2+^, Ca^2+^, and phosphate (P_i_) handling in a precystic mice model [80]. Given the precystic stage of the mice, these changes could not be caused by dilated and cystic tubular structures but were instead attributed to the downregulation of key regulators in Mg^2+^ and Ca^2+^ reabsorption in the TAL (*Cldn16*, *Kcnj1*, *Slc12a1*), DCT (*Trpm6*, *Slc12a3*), and connecting tubule (*Calb1*, *Slc8a1*, *Atp2b4*). Several of these genes are also downregulated in (developing) kidney tissue of *Hnf1b* mutant mice [25, 50, 103]. The presence of cysts in glomerular and tubular nephron structures of ADPKD patients can dramatically impair electrolyte and water homeostasis. However, no association has been described to date between the presence of cysts and hypomagnesemia or other electrolyte phenotypes in ADTKD-HNF1β patients.

Secondly, in vitro and in vivo experiments have shown that HNF1β controls mitochondrial respiration in the PT [15, 61]. Inhibition or KO of HNF1β in a human PT cell line resulted in either downregulation of *Ppargc1a* (important for mitochondrial biogenesis) and altered mitochondrial morphology or ATP reduction and increased glycolysis, respectively [15, 61]. The kidney requires large quantities of ATP to maintain electrochemical gradients across membranes which are particularly important for transcellular ion transport [9]. Given the high energetic demand of the kidneys, the energy deficiency triggered by *HNF1β* defects might influence transport processes in the PT, and potentially TAL and DCT-mediated transport of Mg^2+^, Ca^2+^, and K^+^. Indeed, mutations in the mitochondrial DNA were recently demonstrated to cause a Gitelman-like phenotype of hypomagnesemia and hypokalemia [82].

Finally, over the past years, HNF1β has been implicated in a broad spectrum of pathways ranging from WNT signaling to planar cell polarity and cholesterol synthesis [1, 17, 31]. The role of these pathways in electrolyte transport has never been examined.

## Conclusions and perspectives

Hypomagnesemia, hyperuricemia, and hypocalciuria are common in patients with ADTKD-HNF1β. In subgroups of patients, these electrolyte disturbances are associated with hyperparathyroidism, hypokalemia, and metabolic alkalosis. These clinical findings suggest that the electrolyte disturbances in patients with *HNF1β* defects have a distal tubular origin. Indeed, our literature review demonstrated that HNF1β regulates the expression of genes involved in distal tubule electrolyte transport, including *FXYD2*, *KCNJ16*, *CASR*, and *FXR*. In this review, we propose additional mechanisms that may further contribute to electrolyte disorders. *HNF1β* defects have been demonstrated to impair kidney development, apical-basolateral polarity, tight junction integrity, and cilia development.

The function of HNF1β in kidney physiology has mainly been studied in a wide range of mouse models. Our systematic comparison of all published mouse models identified large differences in phenotypes depending on the genetic defect and strain (Table 1). Complete HNF1β KO may result in different molecular consequences than heterozygous deletions and missense mutations. Consequently, the pathophysiological mechanism of ADTKD-HNF1β may not be captured by most available mouse studies. Moreover, phenotyping of the electrolyte disturbances in HNF1β patients and mouse models is limited, resulting in a knowledge gap in the literature. A more systematic approach is required to associate specific polarity, cilia, or tight junction defects with electrolyte disturbances.

A promising development is the generation of organoid models from patient-derived iPSCs. Recently, kidney organoids were successfully generated from urinary iPSCs of HNF1β patients [53]. Although the current generation kidney organoids are still immature compared with fetal and adult human kidney, these models provide the first patient-derived model to study *HNF1β* defects in kidney development and function [85].

In conclusion, the causes of electrolyte disturbances in ADTKD-HNF1β may partially be beyond direct transcriptional regulation of specific channels and transporters. Further studies should determine which additional pathways contribute to the molecular mechanisms of electrolyte disturbances observed in ADTKD-HNF1β patients. More systematic phenotyping and the development of patient-specific organoid models are essential next steps in HNF1β research.

## Supplementary Information

Below is the link to the electronic supplementary material.

Supplementary file1 (PDF 118 KB)

Supplementary file2 (PDF 109 KB)
