# Correction to: Mechanisms of ion transport regulation by HNF1β in the kidney: beyond transcriptional regulation of channels and transporters

**DOI:** 10.1007/s00424-022-02706-7

**Published:** 2022-05-24

**Authors:** Lotte E. Tholen, Joost G. J. Hoenderop, Jeroen H. F. de Baaij

**Affiliations:** Department of Physiology, Radboud Institute for Molecular Life Sciences, Radboud University Medical Center, P. O. Box 9101, Nijmegen, 6500 HB The Netherlands

**Correction to: Pflügers Archiv - European Journal of Physiology (2022)**

10.1007/s00424-022-02697-5

Originally, the article has been published online with error in Table 1. See below list.

- Na^+^, K^+^, and urea concentrations = Decreased Na^+^, K^+^, and urea urine concentrations.
- Mg^2+^, Na, K^+^, and urea concentrations = Increased total Mg^2+^, Na^+^ and K^+^ urine excretion^d^

In Table 1 footer:

- ^d^ < 12 months of age
- ^e^ In the C57BL/6 N background but not in 129/sv background

The original article has been corrected.

