# Supplementary material for: Mechanisms of ion transport regulation by HNF1β in the kidney: beyond transcriptional regulation of channels and transporters
Source: Pflugers Arch. 2022 May 13;474(8):901–16. doi: 10.1007/s00424-022-02697-5 (PMC9338905; doi:10.1007/s00424-022-02697-5)
Supplement: Supplementary file 1 — Supplementary file1 (PDF 118 KB) [file 424_2022_2697_MOESM1_ESM.pdf]

49 Spadina Ave. Suite 200
Toronto ON M5V 2J1 Canada
www.biorender.com

Confirmation of Publication and Licensing Rights

February 25th, 2022
Science Suite Inc.

Subscription:	*Individual*
Agreement number:	*VW23LUPXWA*
Journal name:	*Pflügers Archiv*

To whom this may concern,

This document is to confirm that Lotte Tholen has been granted a license to use the BioRender content, including icons, templates and other original artwork, appearing in the attached completed graphic pursuant to BioRender's Academic License Terms. This license permits BioRender content to be sublicensed for use in journal publications.

All rights and ownership of BioRender content are reserved by BioRender. All completed graphics must be accompanied by the following citation: “Created with BioRender.com”.

BioRender content included in the completed graphic is not licensed for any commercial uses beyond publication in a journal. For any commercial use of this figure, users may, if allowed, recreate it in BioRender under an Industry BioRender Plan.

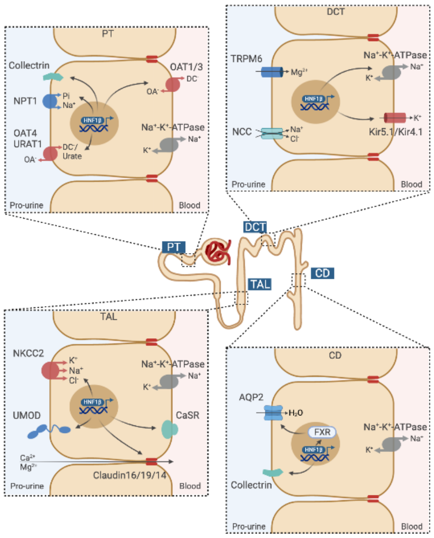

For any questions regarding this document, or other questions about publishing with BioRender refer to our BioRender Publication Guide, or contact BioRender Support at support@biorender.com.
